# What are the statistical implications of treatment non‐compliance in cluster randomized trials: A simulation study

**DOI:** 10.1002/sim.8351

**Published:** 2019-10-03

**Authors:** Mirjam Moerbeek, Sander van Schie

**Affiliations:** ^1^ Department of Methodology and Statistics Utrecht University Utrecht The Netherlands; ^2^ Statistics Netherlands The Hague The Netherlands

**Keywords:** cluster randomized trial, simulation study, treatment non‐compliance

## Abstract

Subjects in randomized controlled trials do not always comply to the treatment condition they have been assigned to. This may cause the estimated effect of the intervention to be biased and also affect efficiency, coverage of confidence intervals, and statistical power. In cluster randomized trials non‐compliance may occur at the subject level but also at the cluster level. In the latter case, all subjects within the same cluster have the same compliance status. The purpose of this study is to investigate the statistical implications of non‐compliance in cluster randomized trials. A simulation study was conducted with varying degrees of non‐compliance at either the cluster level or subject level. The probability of non‐compliance depends on a covariate at the cluster or subject level. Various realistic values of the intraclass correlation coefficient and cluster size are used. The data are analyzed by intention to treat, as treated, per protocol and the instrumental variable approach. The results show non‐compliance may result in downward biased estimates of the intervention effect and an under‐ or overestimate of its standard deviation. The coverage of the confidence intervals may be too small, and in most cases, empirical power is too small. The results are more severe when the probability of non‐compliance increases and the covariate that affects compliance is unobserved. It is advocated to avoid non‐compliance. If this is not possible, compliance status and covariates that affect compliance should be measured and included in the statistical model.

## 1 INTRODUCTION

The cluster randomized trial design is often used in the biomedical, health, and behavioral sciences. With cluster randomized trials complete clusters, such as school classes, households, or general practices are randomized to treatment conditions.^1^, ^2^, ^3^, ^4^, ^5^ The effect of an intervention, relative to a control, can only be estimated without bias when the subjects who are randomized to intervention actually receive the intervention. In practice, non‐compliance often occurs, and in cluster randomized trials, it may not only occur at the level of the subject but also at the level of the cluster. Consider as an example a trial on new dietary guidelines. Non‐compliance at the cluster level occurs when complete families do not comply to these new guidelines, while non‐compliance at the subject level occurs when a few but not all family members do not comply.

There exist various statistical approaches to deal with non‐compliance, for a non‐technical review, see the work of Sagarin and coauthors.^6^ An intention to treat analysis (ITT) is used to estimate the causal effect of treatment assignment, regardless of compliance status. This is done by comparing groups as randomized. Although ITT is generally considered the gold standard, it is often complemented by secondary analyses. An as treated analysis (AT) compares groups on the basis of treatment received. Treating those subjects who did not comply as part of the control group implies the intervention and control group lose comparability on the basis of random assignment. For instance, there may be a higher proportion of less motivated subjects in the control if lower motivation results in higher probability of non‐compliance. A per protocol (PP) analysis only analyzes those subjects who follow their assigned treatment and exclude non‐compliers from the analysis. This is a non‐randomized, observational comparison of groups; any exclusions of subjects from the analysis compromises the randomization and may lead to biased results. AT and PP only estimate the causal effect of treatment received when covariates that affect compliance and the outcome are appropriately adjusted for the work of Hernán and Hernández‐Díaz.^7^ The instrumental variable approach (IV) compares those subjects who would comply with the intervention if they were indeed randomized to the intervention. Hence, it provides an estimate of the complier average causal effect (CACE): the causal effect of intervention in the group of compliers.

ITT, AT, PP, and IV address different research questions as each of them compares different groups.^8^ There exist various recommendations of which approach(es) to use. Hernán and Hernández‐Diaz recommend all trials that lack compliance are analyzed by using ITT and AT and PP.^7^ According to Hartung and Cottrell, ITT should be supplemented by AT.^9^ According to Detry and Lewis, ITT is the cornerstone of the interpretation of randomized controlled trials, but they argue that in some cases, PP may be helpful, for instance, in pharmaceutical trials with too high‐dose levels that patients cannot adhere to because of intolerable adverse effects.^10^ On the other hand, Welsh questions the ubiquitous adherence to ITT.^11^ In the most recent version of the CONSORT statement, the specific request for ITT has been dropped in favour of a clear description of exactly who was included in each analysis.^12^ Wu et al conclude that neither ITT nor PP can always guarantee the validity of the conclusion in non‐inferiority trials.^13^

Given the variety of approaches to deal with non‐compliance and recommendations of which approach to use, it is important to understand how each of them performs. Only then, a motivated choice can be made for a trial at hand. Various simulation studies have been conducted to study the performance of these approaches. Sanchez and Chen compare ITT and PP and recommend a hybrid of the two.^14^ Bang and Davis compare ITT, AT, PP, and IV and conclude ITT and IV are not perfect and can be problematic in some situations.^15^ Jo compares statistical power for ITT and CACE and shows outcome distributions and pretreatment covariates are sources that may increase the difference in power between these two approaches.^16^ Ye and co‐authors give a decision scheme for choosing the best approach for different non‐compliance scenarios.^17^ Other authors used derived analytical expressions to compare approaches. Kim shows that increasing sample sizes are needed as the expected proportion of non‐compliers increases.^18^ McNamee concludes that ITT is not always biased towards the null hypothesis, while AT and PP are.^19^ Sheng and Kim studied ITT of equivalence trials and also conclude non‐compliance does not always favour the null hypothesis.^20^

The common feature of these studies is that they focus on trials with subject randomization. Non‐compliance in cluster randomized trials has been studied for encouragement trials^21^, ^22^ and trials with survival outcomes.^23^ Attention has also been paid to the estimation of CACE^24^, ^25^ and sample size issues.^26^, ^27^

It is important to understand how ITT, AT, PP, and IV perform for cluster randomized trials. Although AT and PP do not always estimate causal effects, they are indeed used. A systematic review of 123 cluster randomized trials showed non‐compliance was reported by 56 trials (46%). Of these, 19 trials adjusted for non‐compliance: 15 used PP and 4 used AT. None of the studies reported an estimate for CACE.^28^

This contribution presents the results of an extensive simulation study on the statistical implications of non‐compliance in cluster randomized trials. It does not only include ITT and IV but AT and PP as well; hence, it extends previous simulation studies that are restricted to ITT and CACE.^29^, ^30^ Our simulation study uses realistic values for cluster size and intraclass correlation coefficient. Non‐compliance may occur at the subject or cluster level and the probability of non‐compliance depends on a covariate that is measured at the subject or cluster level. We study scenarios where the average probability of non‐compliance ranges from 0 to 0.5. Two analyses are performed: one that does and another one that does not include this covariate. As such, we mimic studies with observed and unobserved covariates. We consider trials that carefully restrict access to the intervention; hence, we only consider compliers and never‐takers. Four performance measures are used: mean estimate, efficiency, coverage of confidence intervals, and statistical power. On the basis of the results of our simulation, researchers can make a motivated choice for which approach(es) to use for their cluster randomized trial.

## 2 STATISTICAL APPROACHES TO DEAL WITH NON‐COMPLIANCE

**Figure 1:**
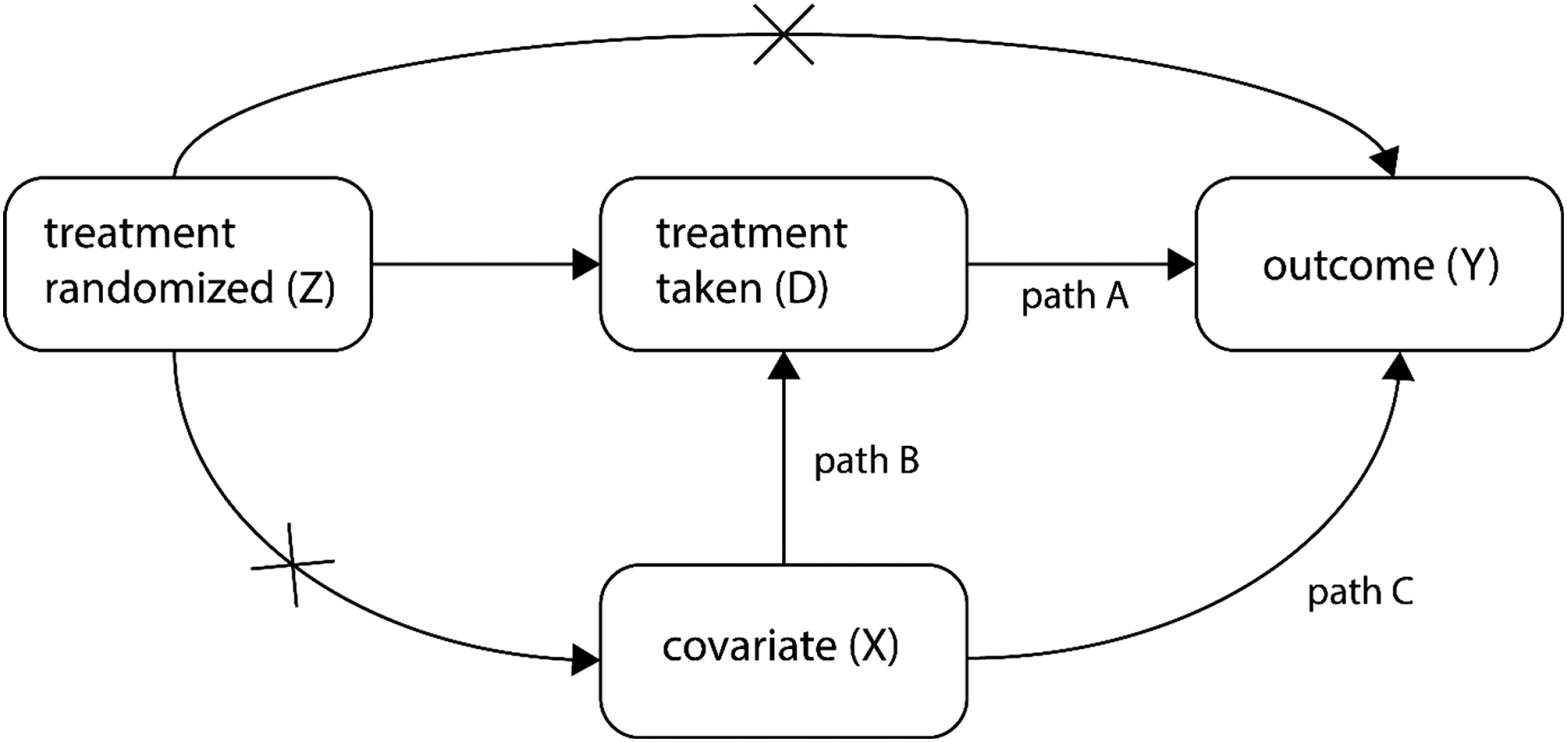
Graphical representation of non‐compliance model. The two paths that are crossed‐out represent relationships that are equal to zero

Figure 1 is used to explain the different statistical approaches to deal with non‐compliance. For a more extensive explanation, see the work of Jo.^16^ Randomization to treatment conditions occurs at the cluster level and *Z*_*j*_ = 0 if cluster *j* is randomized to the control and *Z*_*j*_ = 1 if it is randomized to the intervention. It is assumed there is no interference between clusters, meaning that a subject's outcome does not depend on outcomes of subjects in other clusters. Each subject is exposed to either the intervention or control, but not to both; hence, only one outcome can be observed. A subject's potential outcome under a given condition that happened to materialize is precisely the outcome experienced by that subject (consistency assumption). Each subject has two potential outcomes, one for each condition. These are the outcomes that would have been observed had the subject been randomized to the intervention or the control. Potential outcomes are used to define the assumptions under which the compliers average causal effect is identified and are an important part of Rubin's causal model.^31^

The treatment received by subject *i* in cluster *j* is denoted *D*_*ij*_ and depends on the treatment randomized *Z*_*j*_ and observed and/or unobserved covariates that may vary between or within clusters. Treatment randomized *Z*_*j*_ is the instrumental variable and we assume *cov*(*Z*_*j*_, *D*_*ij*_) ≠ 0 (ie, the assumption of instrument relevance). We consider one covariate *X*_*ij*_ in our simulation. Such a covariate may be measured at the level of the subject, such as age or gender, or at the level of the cluster, such as hospital size. Both the covariate and treatment received have an effect on the subject‐level outcome *Y*_*ij*_. The arrows in Figure 1 denote causal relationships. The two paths that are crossed‐out imply treatment randomized only has an indirect effect on the outcome, through treatment received, and treatment randomized does not have an effect on the covariate. This implies covariates should be measured at baseline or should not be subject to change during the course of the trial, such as gender and age. A covariate such as motivation is not suitable since it may change as a result of treatment assigned.

Based on treatment randomized *Z*_*j*_ and treatment received *D*_*ij*_, four compliance strata can be defined. Compliers always comply with the treatment they are randomized to: *D*_*ij*_(*Z*_*j*_) = *Z*_*j*_ for *Z*_*j*_ = {0,1}. Never‐takers never receive the intervention: *D*_*ij*_(*Z*_*j*_ = 0) = *D*_*ij*_(*Z*_*j*_ = 1) = 0, while always‐takers always receive the intervention *D*_*ij*_(*Z*_*j*_ = 0) = *D*_*ij*_(*Z*_*j*_ = 1) = 1. Defiers receive the opposite of the treatment they were randomized to *D*_*ij*_(*Z*_*j*_) = 1 − *Z*_*j*_ for *Z*_*j*_ = {0,1}. The four classes are needed to define the assumptions under which the compliers average causal effect is identified (see later in this section). Note that in the case of always‐takers and defiers, subjects who are randomized to the control somehow have access to the intervention. In our simulation study, we only consider compliers and never‐takers, in other words, we exclude the possibility *D*_*ij*_(*Z*_*j*_ = 0) = 1.

In case non‐compliance is absent *D*_*ij*_ = *D*_*j*_ = *Z*_*j*_ and the effect of intervention is estimated from the following model

$$Y_{\text{ij}}=\beta_{0}+\beta_{1}D_{j}+\beta_{2}X_{\text{ij}}+u_{j}+e_{\text{ij}},$$

where *u*_*j*_∼*N*(0, *τ*^2^) and *e*_*ij*_∼*N*(0, *σ*^2^) are between‐ and within cluster random effects, and these are assumed independent of each other. This is a multilevel or mixed‐effects model since it explicitly takes into account between‐cluster variation.^32^, ^33^, ^34^, ^35^ The intraclass correlation coefficient (ICC) quantifies the proportion of variance that is located at the cluster level *ρ* = *τ*^2^/(*τ*^2^+*σ*^2^). The fixed regression coefficients *β*_0_ and *β*_1_ are the expected outcome in the control condition and the expected effect of treatment, respectively. The estimate of *β*_1_ is an unbiased estimate of the causal effect of treatment if assignment to treatments is done at random, there is no interference between the clusters in the trial and the consistency assumption holds. The first assumption implies treatment assignment does not depend on the covariates; the second assumption implies a subject's potential outcomes are independent of the outcomes from subjects in other clusters.

It will often occur *D*_*ij*_ ≠ *Z*_*j*_ and non‐compliance has to be adjusted for if it is guided by the trial protocol or objectives. ITT does not adjust for non‐compliance but analyzes all subjects as they were randomized, using the model *Y*_*ij*_ = *β*_0_+*β*_1_*Z*_*j*_+*β*_2_*X*_*ij*_+*u*_*j*_+*e*_*ij*_. In other words, the causal estimand for an ITT analysis is the causal effect of treatment assignment. This implies that trials with different adherence patterns may provide different estimates of the causal effect of treatment received.

AT and PP do adjust for non‐compliance by analyzing subjects according to treatment received based on the model *Y*_*ij*_ = *β*_0_+*β*_1_*D*_*ij*_+*β*_2_*X*_*ij*_+*u*_*j*_+*e*_*ij*_. The difference is that AT includes all subjects and PP restricts to those who comply to their assigned treatment (*D*_*ij*_ = *Z*_*j*_). The causal estimand of these two approaches is the causal effect of treatment received, provided that covariates that affect compliance and the outcome are adequately adjusted for.

The causal estimand for the IV approach is CACE, the causal effect of treatment in the group of compliers. It is identified when we make three sets of assumptions. The technical assumptions are those of no interference between clusters and consistency as explained at the beginning of this section. The instrumental variable assumptions are those of random assignment, instrument relevance and exclusion restriction, where the latter states that the potential outcomes of non‐compliers in the intervention and control condition are the same regardless of treatment assignment. The point identification assumption states that there are no defiers (monotonicity assumption). Finally, it should be noted that CACE can only be estimated when the proportion non‐compliers is less than one: there are at least some subjects who comply with treatment randomized.

As compliance status cannot be observed in the control condition, advanced statistical techniques are needed to estimate CACE. With the IV approach, intervention effects are adjusted by considering the non‐compliance rate.^36^, ^37^, ^38^ It uses two‐stage least squares to estimate the complier average causal effect. First, treatment received *D*_*ij*_ is regressed on the instrumental variable treatment randomized *Z*_*j*_ to get $\hat{E}\left(D_{\text{ij}}|Z_{j}\right)$. The model equation is *D*_*ij*_ = 1{*α*_0_+*α*_1_*Z*_*j*_+*e*_1*ij*_ > 0}, where 1{·} is the indicator function that is equal to 1 if its argument is true and 0 otherwise. Second, the outcome *Y*_*ij*_ is regressed on $\hat{E}\left(D_{\text{ij}}|Z_{j}\right)$, with model equation $Y_{\text{ij}}=\beta_{0}+\beta_{1}\hat{E}\left(D_{\text{ij}}|Z_{j}\right)+e_{2\text{ij}}$. The compliers' average causal effect is estimated by ${\hat{\beta }}_{1}$.^24^, ^39^ This can be done by using the package ivpack in R and by using the Huber‐White standard errors that are robust for clustering.^40^ That is true given a sufficient number of clusters, but not when the number of clusters is much below 40. It should be noted that two‐stage least squares estimation of CACE can be problematic when the association between the instrument *Z*_*j*_ and endogenous variable *D*_*ij*_ is weak, and in that case, the type I error rate can be much larger than the nominal value. Staiger and Stock propose a rule of thumb that states that the first‐stage partial F statistic for testing that the effect of the instrument is zero should be 10 or larger.^41^

## 3 DESIGN OF SIMULATION STUDY

A simulation study was carried out to study the effects of non‐compliance in cluster randomized trials. In order to run, the simulations R version 3.5.0 was used.^42^ For the ITT, AT, and PP approach, the function lme, which is part of the package nlme, was used for parameter estimation and null hypothesis testing.^43^ Restricted maximum likelihood was used to obtain variance component estimates. This estimation approach was chosen over full‐information maximum likelihood, because restricted maximum likelihood generates less biased variance components, particularly when the number of clusters is small. For IV, no multilevel regression is used but standard regression assuming independence between subjects. The clustering is subsequently taken into account by using robust clusters standard errors. The functions ivreg and cluster.robust.se, which are part of the package ivpack, were used for this.^44^

### 3.1 Factors and levels

To investigate the effects of non‐compliance, three fixed factors and four factors with various levels were used (see Table 1 for an overview). The number of combinations of all factor levels was 3*3*2*6 = 108. Henceforward, each combination will be called a simulation condition. First of all, the casual effect size of treatment received was fixed at *β*_1_ = 0.2 (path A in Figure 1). Furthermore, the intraclass correlation coefficient was *ρ* = 0.025, 0.05, or 0.10. Note that this is a conditional intraclass correlation coefficient since it is based on a model that includes predictors *D*_*ij*_ and *X*_*ij*_. The chosen values span a wide range of realistic values that are often found in cluster randomized trials in school settings and primary and secondary care, see table 11.1 of the work Moerbeek and Teenstra.^45^ The cluster size was *n*_1_ = 10, 20, or 50, which could represent sports teams, school classes, and hospital departments, respectively. The cluster size was kept constant across clusters. For each simulation condition the number of clusters *n*_2_ was calculated from

$$n_{2}=4\left(\sigma^{2}+\tau^{2}\right)\frac{1+\left(n_{1}-1\right)\rho }{n_{1}}{\left(\frac{z_{1-\frac{\alpha }{2}}+z_{1-\beta }}{\beta_{1}}\right)}^{2},$$

with total variance *σ*^2^+*τ*^2^ = 1.45 The power level 1 − *β* and type I error rate α were fixed at 0.8 and 0.05, respectively, and two‐sided testing was done. This value of *n*_2_ was rounded upwards to the nearest integer. This caused the power to be slightly higher than 0.80 and power levels could slightly differ between simulation conditions. The possibility of non‐compliance is usually not taken into account when calculating the desired number of clusters. So these calculations were done under the assumption that all clusters and subjects would comply. The number of clusters needed in the simulation ranged from 38 to 152 and was sufficiently large for the Huber‐White standard errors to be robust to clustering.

**Table 1:** Factors and levels in the simulation study

Factor	Level(s)
Treatment effect (*β*_1_)	0.2
Intraclass correlation coefficient (*ρ*)	0.025, 0.05, 0.1
Cluster size (*n*_1_)	10, 20, 50
Number of clusters (*n*_2_)	such that power is 80%
Level of non‐compliance ( ${\bar{P}}_{\text{NC}}$)	cluster, subject
Average probability of non‐compliance	0, 0.1, 0.2, 0.3, 0.4, 0.5
Covariate effect (*β*_2_)	0.2

The focus of the paper is the effect of non‐compliance. Non‐compliance could occur at the cluster level or subject level. ${\bar{P}}_{\text{NC}}$, the average probability of non‐compliance, was set at 0, 0.1, 0.2, 0.3, 0.4, or 0.5. The actual probability of non‐compliance was varied based on the value of a covariate *X*_*ij*_ (path B in Figure 1). For both cluster and subject level non‐compliance, the actual probability of non‐compliance was calculated using the quantiles from the standard normal distribution *z*. If *X*_*ij*_ < *z*_1/3_ then $P_{\text{NC}}=0.5{\bar{P}}_{\text{NC}}$, if *z*_1/3_ < *X*_*ij*_ < *z*_2/3_ then $P_{\text{NC}}={\bar{P}}_{\text{NC}}$, and if *X*_*ij*_ > *z*_2/3_ then $P_{\text{NC}}=1.5{\bar{P}}_{\text{NC}}$. For the simulations with non‐compliance at the cluster level, *X*_*ij*_ was generated from the standard normal distribution. For the simulations with non‐compliance at the subject level, *X*_*ij*_ had both a within and between component. Both of these components were normally distributed with mean zero. The total variance of *X*_*ij*_ was set to 1 and the ICC was chosen to be equal to that of the outcome *Y*_*ij*_. The covariate *X*_*ij*_ did not only have an indirect effect on the outcome *Y*_*ij*_ through treatment received, but also a direct effect (path C in Figure 1). The size of this direct effect is *β*_2_ = 0.2.

For each simulation condition, the number of generated data sets was set at 5000. For each data set the outcome *Y*_*ij*_ was generated from the model with treatment received *D*_*ij*_ and the covariate *X*_*ij*_ as predictors: *Y*_*ij*_ = *β*_0_+*β*_1_*D*_*ij*_+*β*_2_*X*_*ij*_+*u*_*j*_+*e*_*ij*_. This implies the outcome data for non‐compliers in the treatment condition were generated under the same model as for subjects in the control condition. Each data set was analyzed twice: once with and once without the covariate *X*_*ij*_ included in this model. Note that the model that ignores the covariate is basically a misspecified model, and we may expect worse results than for the model that does include the covariate.

### 3.2 Criteria for evaluation

Four criteria were studied to evaluate the effect of non‐compliance. These are evaluated over all 5000 generated data sets per simulation condition. The first criterion is the mean treatment effect estimate. For each approach, this mean estimate is compared to its target estimand. For AT, PP, and IV, this is equal to the effect of treatment received: *β*_2_ = 0.2. The effect of treatment assignment is lower than the effect of treatment received and depends on the average probability of non‐compliance: $\beta_{2}\left(1-{\bar{P}}_{\text{NC}}\right)$. Hence, for ITT, we compare the mean estimate to this value. The second criterion is the standard deviation of the treatment effect estimates. The aim is to study how the standard error changes if the average probability of non‐compliance increases. The third is the coverage of the 95% confidence interval for the treatment effect; for each of the four approaches, it is calculated by taking the proportion of confidence intervals, which contain the population value of the target estimand of that approach. The limits of the confidence intervals are calculated as ${\hat{\beta }}_{1}\pm 1.96*\text{se}\left({\hat{\beta }}_{1}\right).$ The fourth is the empirical power for the test on treatment effect, which is calculated by taking the proportion of generated data sets for which the treatment effect was significant. We aim to study how the power is influenced by the average probability of non‐compliance.

In addition, for each generated data set, we calculated the partial F statistic to test the effect of the instrumental variable.

## 4 RESULTS OF SIMULATION STUDY

For all generated data sets, the partial F test was (much) larger than 10, meaning that the strength of the instrumental variable is sufficiently large.

**Figure 2:**
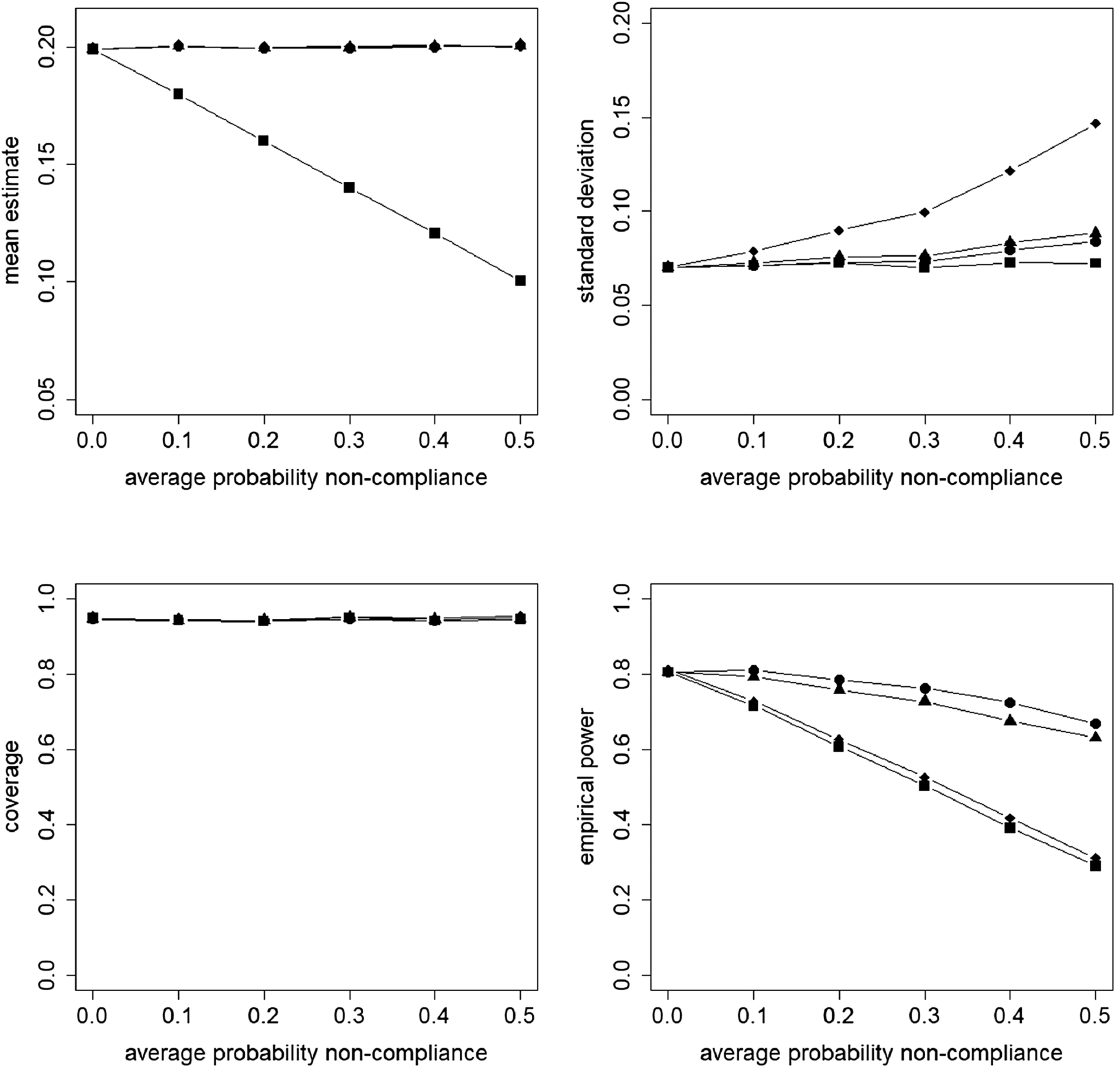
Mean estimate, standard deviation, coverage of confidence interval, and empirical power as a function of the average probability of non‐compliance. The non‐compliance is at the cluster level. The covariate X is included in the model. 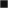 = intention to treat; 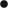 = as treated; 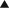 = per protocol; 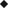 = instrumental variables

**Figure 3:**
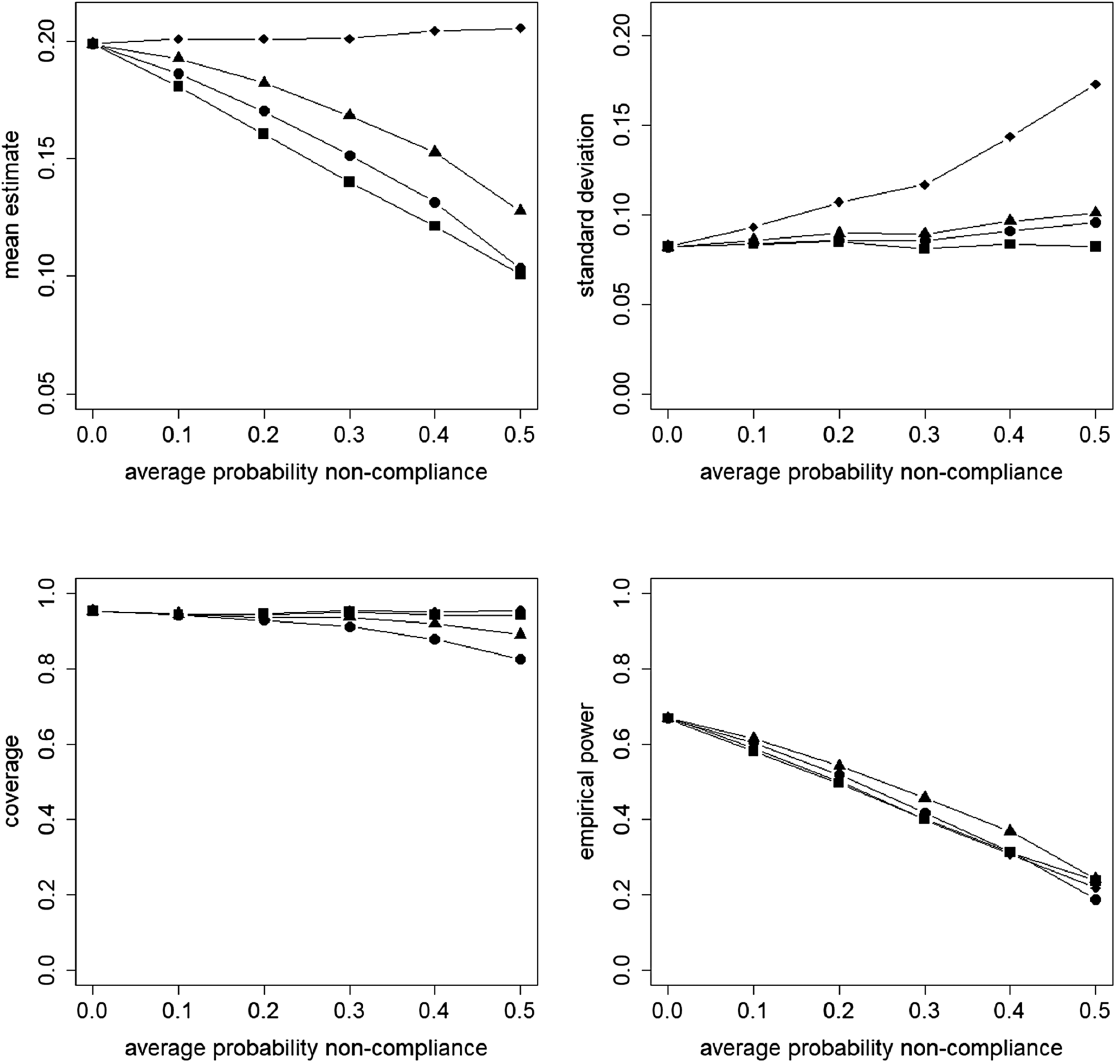
Mean estimate, standard deviation, coverage of confidence interval, and empirical power as a function of the average probability of non‐compliance. The non‐compliance is at the cluster level. The covariate X is not included in the model. 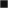 = intention to treat; 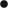 = as treated; 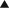 = per protocol; 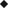 = instrumental variables

**Figure 4:**
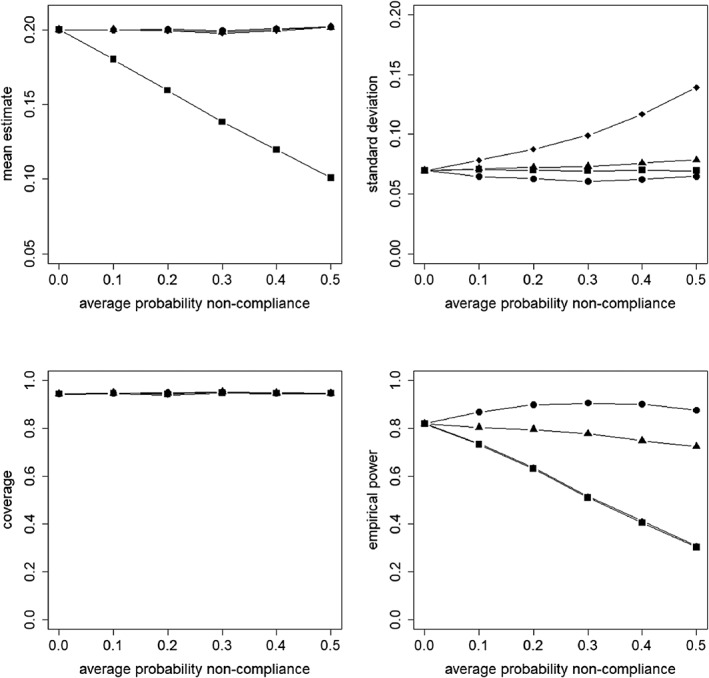
Mean estimate, standard deviation, coverage of confidence interval, and empirical power as a function of the average probability of non‐compliance. The non‐compliance is at the subject level. The covariate X is included in the model. 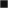 = intention to treat; 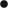 = as treated; 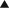 = per protocol; 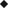 = instrumental variables

**Figure 5:**
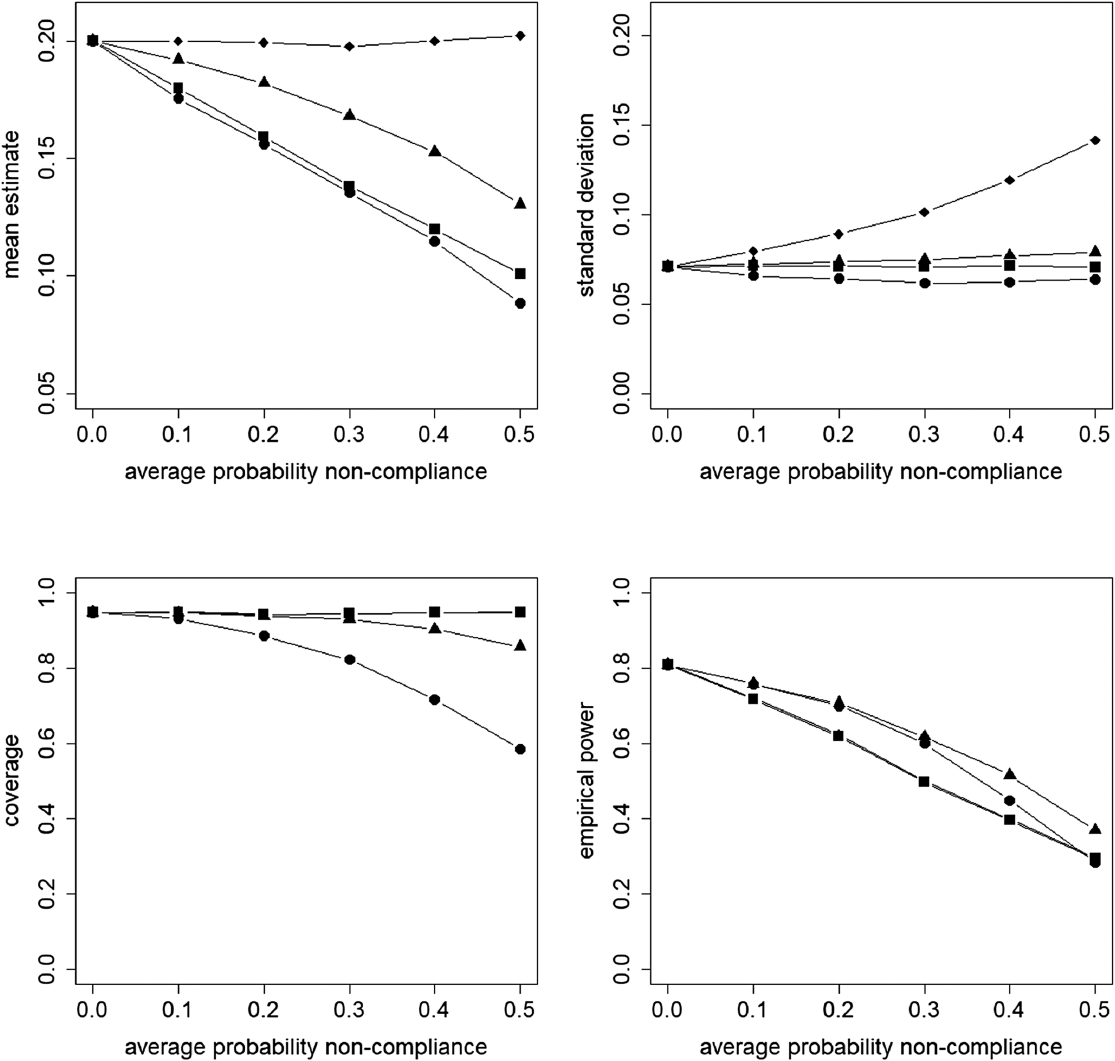
Mean estimate, standard deviation, coverage of confidence interval, and empirical power as a function of the average probability of non‐compliance. The non‐compliance is at the subject level. The covariate X is not included in the model. 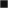 = intention to treat; 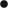 = as treated; 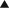 = per protocol; 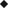 = instrumental variables

Figures 2, 3, 4, 5 present the results for the simulation conditions in which the ICC was 0.05 and cluster size was 20. The results of the conditions with other ICC and cluster size values were similar and any differences are discussed at the end of this section. The results for all conditions are presented in the supplementary material.

### 4.1 Non‐compliance at cluster level, covariate included in the model

Figure 2 presents the results for non‐compliance at the cluster level for the analysis with the covariate included in the model. For the ITT approach, the mean estimate is unbiased for its target estimand, which has a population value of $\beta_{2}\left(1-{\bar{P}}_{\text{NC}}\right)$. We observe the estimated causal effect of treatment assignment linearly decreases when the average probability of non‐compliance increases. For the three other approaches AT, PP, and IV, the mean estimate is near the true value of its target estimand for all probabilities of non‐compliance. It should be noted that although these target estimands have different interpretations, they are of the same value *β*_2_ = 0.2.

For ITT, the standard deviation is hardly influenced by the probability of non‐compliance. For AT, PP, and IV, the standard deviation increases when the probability of non‐compliance increases, especially so for IV.

For all four approaches, the coverage of the confidence intervals is near 95% for all probabilities of non‐compliance.

Finally, for all four approaches, the empirical power is equal to the desired power level of 0.8 when non‐compliance is absent, but decreases when the probability of non‐compliance increases. The decline in power is less severe for the AT and PP approaches, compared to the ITT and IV approaches. The decreasing power for ITT results from the fact that the causal effect estimate of treatment assignment decreases with increasing probability of non‐compliance. For the other approaches, it results from an increase in standard deviation.

### 4.2 Non‐compliance at cluster level, covariate not included in the model

In Figure 3, the results for non‐compliance at the cluster level are presented for the analysis that ignores the covariate. It shows that for ITT and IV, the mean treatment effect estimate is near the population value of the target estimand for any value of the probability of non‐compliance. For AT and PP, the mean estimate decreases when the probability of non‐compliance increases, where AT shows the highest decrease. The results are different from the model where the covariate is included (see Figure 2), for which each approach estimated its target estimand without bias.

The results for the standard deviation are quite similar to the model where the covariate is included (Figure 2). So for ITT, it is hardly influenced by the probability of non‐compliance, but for AT, PP, and IV, the standard deviation increases when the probability of non‐compliance increases, especially so for IV. In addition, compared to the model that includes the covariate, the standard deviation is slightly higher. This can be explained by the fact that the covariate on the between level explains part of the variance on that level, and that variance in its turn has an effect on the standard deviation. So not including such a covariate in the analysis causes the standard deviation to be higher.

For ITT and IV, the coverage of the confidence interval is hardly influenced by the probability of non‐compliance and it is near 95%. For AT and PP, the coverage decreases when the probability of non‐compliance increases, and the undercoverage is a result of an underestimate of the target estimand. This is in contrast with the model that includes the covariate (see Figure 2). In that model, the coverage was near 95% for all approaches.

The empirical power decreases when the probability of non‐compliance increases. For AT and PP, this is explained by an underestimate of their causal estimand, for IV, by an increased standard error. For ITT, the causal estimate of treatment assignment decreases with increasing probability of non‐compliance; hence, the power decreases. In contrast to the model that includes the covariate (Figure 2), the decrease in power is equally severe for all approaches. Also, the decrease is more severe than for the ITT and IV approaches in the model in which the covariate is included. A final remark is that even when there is no non‐compliance, the power level is below .80. As the covariate is not included in the model, a higher standard deviation is obtained, which results in a lower power level.

### 4.3 Non‐compliance at subject level, covariate included in the model

Figure 4 presents the results for non‐compliance at the subject level and an analysis that includes the covariate in the model. For each approach, the mean treatment effect estimate is about equal to the population value of its target estimand. Again, it should be noted that although the target estimands of AT, PP, and IV have different interpretations, they are of the same value *β*_2_ = 0.2.

For the ITT approach, the standard deviation is hardly influenced by the probability of non‐compliance. In contrast, for PP and IV, the standard deviation increases when the probability of non‐compliance increases, especially so for IV. On the other hand, the standard deviation of AT decreases when the probability of non‐compliance increases to 0.3, but increases at higher probabilities. This is not the case for AT in the cluster non‐compliance setting, where the standard deviation decreases monotonically (see Figure 2). The reason for this difference is that for AT, non‐compliers are analyzed as if they were in the control condition. In the cluster non‐compliance setting, this implies that those clusters that did not comply were analyzed as if in the control condition and so were all subjects in these clusters. In such a case, a between‐cluster comparison of treatment conditions is made. In the subject non‐compliance setting, non‐compliance implies that in the clusters that were randomized to treatment, there were subjects who complied with treatment but also subjects who did not and were analyzed as in the control. In these clusters, a within comparison of treatments is made, which results in higher efficiency, but only until the probability of non‐compliers is 0.3. When this probability further increases, the number of subjects to be analyzed as control becomes high as compared to the number of subjects that comply with treatment. So then, the design becomes too much unbalanced and this causes the standard deviation to increase at higher values of the probability of non‐compliance.

For each approach, the coverage of confidence intervals is about 95% and is hardly affected by the probability of non‐compliance.

For all approaches, the power is at about 0.8 when all subjects comply. For ITT and IV, the decrease of power is rather severe and linear over the probabilities of non‐compliance. For ITT, the decrease is explained by the fact that the causal effect estimate of treatment assignment becomes smaller with increasing probability of non‐compliance, for IV, it is caused by the increasing standard error. For PP, there is only a slight decrease, caused by the increasing standard error. In contrast, with the AT approach, the power increases when the probability of non‐compliance increases to 0.3, and increases at higher levels of the probability of non‐compliance. This is a result of the behaviour of the standard deviation as explained above.

### 4.4 Non‐compliance at subject level, covariate not included in the model

Figure 5 presents the results for non‐compliance at the subject level for the analysis that ignores the covariate. It shows that, as compared to the model where the covariate is included (see Figure 4), the mean treatment effect estimate for AT and PP deviates from the population value of its target estimand, especially so when the probability of non‐compliance increases. The decrease is worst for AT. For ITT and IV, the mean treatment effect estimate is constant and near the population value of their target estimands.

The results for the standard deviation are practically the same as when the covariate is included in the model (see Figure 4). So for ITT, it is hardly influenced by the probability of non‐compliance, for PP and IV, the standard deviation increases when the probability of non‐compliance increases, especially so for IV. For AT, the standard deviation decreases until the probability of non‐compliance is 0.3, then it increases slightly. It should further be mentioned that the models with and those without the covariate have the same values of the standard deviation. This is explained by the fact that a subject‐level covariate explains part of the within cluster variance but causes the between cluster variance to increase once it is added to the model.^35^ In our simulation study, the two effects outweigh each other.

There are some differences in coverage compared to the model in which the covariate is included (see Figure 4). In that model, a coverage near 95% was observed for all approaches, whereas in the model without the covariate, the coverage for AT and PP decreases when the probability of non‐compliance increases. The decrease is worst for AT and less severe for PP and for both approaches, it is a result of the underestimate of the target estimand. For ITT and IV, the coverage is hardly influenced by the probability of non‐compliance and about 95%.

In contrast to the model in which the covariate is included (see Figure 4), for all approaches, the power decreases when the probability of non‐compliance increases. This decrease is a result of the underestimated treatment effect (AT and PP) or overestimated standard deviation (IV). For ITT, it is explained by the fact that the estimate of the causal effect of treatment assignment becomes smaller when the probability of non‐compliance increases.

### 4.5 The effect of the level of non‐compliance

In general, the results in Figures 2 and 4 show that the level of non‐compliance has a minor effect when the covariate is included in the model. The main difference is the behaviour of the standard deviation for AT, and as a result of that the effect of non‐compliance on empirical power. The explanation for this difference was given above.

For the models in which the covariate is not included (see Figures 3 and 5), some differences are found for the mean treatment effect estimate and the coverage of the confidence intervals. For cluster non‐compliance, the causal effect estimate of treatment received based on AT is higher than the causal effect estimate of treatment assignment based on ITT, whereas for subject non‐compliance, the opposite holds. Furthermore, the detrimental effect of the probability of non‐compliance on the coverage of confidence intervals for AT is more severe with subject non‐compliance than with cluster non‐compliance.

### 4.6 The effect of cluster size and intraclass correlation coefficient

Figures 2–5 present results for ICC *ρ* = 0.05 and cluster size *n*_1_= 20. In this section, the results for all values of *ρ* and *n*_1_ are summarized and noteworthy findings are discussed. The extensions to Figures 2–5 can be found in the online supplementary material.

For each ICC and for each cluster size, ITT and IV estimate their target estimand without bias, regardless of whether the covariate is included, but AT and PP only provide an unbiased estimate of treatment received when the covariate is included in the model. For cluster non‐compliance and an analysis that ignores the covariate, the estimate of the causal effect of treatment received based on AT is higher than the estimate of the causal effect of treatment assignment based on ITT. For subject non‐compliance and an analysis that ignores the covariate, the estimate of the causal effect of treatment received based on AT may be lower than the estimate of the causal effect of treatment assignment based on ITT, especially so when the cluster size and/or intraclass correlation coefficient and/or average probability of non‐compliance are large.

For IV, the standard deviation always increases with increasing probability of non‐compliance. For cluster non‐compliance and an analysis that does not include the covariate, the increase of the standard deviation of IV increases with increasing cluster size but only when the ICC is 0.025 or 0.05. Besides this, there is also the behaviour of the standard deviation with the AT approach when there is non‐compliance at the subject level. This behaviour is more extreme when the ICC and/or cluster size increase.

AT and PP cause undercoverage of the confidence interval when the covariate is not in the model. Cluster size and ICC do not affect the undercoverage of AT and PP in case of cluster non‐compliance, but they do so for AT in case of subject non‐compliance. For the latter, the undercoverage for the AT approach is more severe when cluster size and/or ICC increase. Finally, the ITT and IV approaches give a coverage near 95% for both cluster and subject non‐compliance, irrespective of whether the covariate is included.

For all approaches, the power decreases with the average probability of non‐compliance when there is non‐compliance at the cluster level. When the covariate is included in the model, the largest reduction in power is for the ITT and IV approaches and cluster size and ICC do not influence this much. When the covariate is not included in the model, the power of all approaches is lower than the preferred level of 0.8, even when there is no non‐compliance. This is influenced by cluster size and ICC: lower power is observed for higher cluster size and lower ICC. With subject non‐compliance, the power decreases with increasing probability of non‐compliance, except for AT. When the covariate is included in the model, the power of AT increases at lower probabilities of non‐compliance but may decrease when this probability becomes higher. This tendency is stronger when cluster size and/or ICC are large. In case the covariate is not included in the model, this behaviour is only observed for *ρ* = 0.1 and cluster size *n*_1_= 50. For smaller values of these two design factors, the power always increases with increasing probability of non‐compliance and this is explained by the underestimated treatment effect.

## 5 DISCUSSION

The results of our simulation study show that non‐compliance may result in severely biased results. AT and PP may underestimate the population value of their target estimand when the covariate is not included in the model; the underestimate becomes more severe when the probability of non‐compliance increases. In most cases, the standard error of AT, PP, and IV increases with the probability of non‐compliance. For moderate probabilities of non‐compliance at the subject level, PP may result in a smaller standard deviation and an increased power in case the covariate is included in the model. In general, the results get worse when the probability of non‐compliance increases and/or when the covariate that influences the probability of non‐compliance is not included in the statistical model.

As we mentioned in the introduction, our simulation study extends previous simulation studies by Jo and co‐authors^29^, ^30^ as it does not only take ITT and CACE into account but also AT and PP. There are other differences between our simulation and those by Jo et al. We considered a range of probabilities of non‐compliance while Jo et al considered one value. We used a covariate to generate compliance status, while Jo et al generated compliance status based on an intraclass correlation for compliance, for which they used a wide range. They found that power for ITT decreased with increasing value of the ICC for compliance, and that coverage of confidence intervals for CACE decreased with an increasing value of the ICC for compliance. In other words, an increasing ICC for compliance has a detrimental effect.

We conclude that avoiding non‐compliance in cluster randomized trials is the best approach. It is therefore important to study means to motivate subjects to comply to treatment. Compliance may be better with a preferred treatment. Marcus and coworkers propose a doubly blind randomized preference trial, which is a hybrid of a randomized and non‐randomized design.^46^ Subjects are first randomized to randomized assignment or choice assignment. Those in the first group are subsequently randomly assigned to treatments and those in the latter receive the treatment of their choice. In a recent cluster randomized trial in mental health care, higher compliance rates were indeed observed among those families that were offered treatment of their choice.^47^

If compliance rates cannot be increased, then at least one should try to measure each cluster's and subject's compliance status and identify and measure covariates that influence the probability of non‐compliance and include these as covariates in the statistical model for data analysis, see also the work of Ten Have et al.^48^ Measuring compliance status is important because adjusting for non‐compliance can only be done if compliance status is known. Unfortunately, subjects in a trial are often dishonest about their compliance status.^49^ It is therefore important to develop methods to detect non‐compliance, such as biomarkers, monitoring techniques, and subject registries.^49^ One can think of devices to measure drug levels in blood or urine, or smart watches to measure physical activity. In some trials, compliance status may be easier to measure, such as whether a participant attended a sufficient amount of meetings with a psychotherapist. In addition to that, it is important that compliance rates are clearly reported with the aim to inform coordinators of future trials on anticipated non‐compliance rates. The CONSORT statement for cluster randomized trials recommends using a flow diagram for communicating the flow of participants and clusters throughout the trial.^12^ Three recent reviews show that reporting of non‐compliance rates is often inadequate.^50^, ^51^, ^52^

As our simulation shows, avoiding covariates that influence compliance status may have a strong impact on the results of a cluster randomized trial. It is therefore important such covariates are identified, measured, and included in the statistical model. We acknowledge that it may not always be clear that covariates should be taken into account and the selection of such covariates may depend on the investigators' experience and expectations. For instance, one may expect immigrants to have lower compliance status than natives when the intervention relies on interpersonal communication in a country's official language(s). Likewise, illiterates may experience higher non‐compliance rates than literates when the intervention consists of (mainly) written material in self‐help trials. It is highly important that papers report covariates that have an effect on compliance, again to facilitate the design of future trials.

Although our simulation was extensive in the sense that it took many realistic scenarios into account, its shortcomings should not be ignored. First, it used a simple setting in the sense that the target estimands of AT, PP, and IV were of the same value, which is not always the case in practice. There is a possibility that non‐compliers may have better outcomes when assigned to the control than to the intervention, for instance, because poorly complying subjects may feel embarrassed or are demoralized by failing to comply with the treatment. In such cases, the target estimands of AT, PP, and IV may be of a different values. Second, our simulation did not take into account the possibility of non‐compliance at the cluster and subject level simultaneously. Third, it only included a covariate at the level at which non‐compliance occurs, while in practice, the covariate may be at the other level or there may be covariates at both levels. Fourth, it may also occur non‐compliance is only partial. Two recent simulation studies focused on partial non‐compliance in an individually randomized trial.^17^, ^53^ They either used the degree of non‐compliance as predictor variable or dichotomized non‐compliance by using a threshold above, which a subject is considered a non‐complier. It would be interesting to repeat these simulation studies in the setting of a cluster randomized trial. Furthermore, our simulation showed that in most cases, empirical power is inversely related to the average probability of non‐compliance. It is worthwhile to extend the current knowledge on sample size determination and derive mathematical expressions for the relation between sample size and power.^26^, ^27^ Other directions for future research include non‐compliance in cluster randomized trials with missing outcome data^54^, ^55^, ^56^ and trials with partial nesting.^57^

## 6 CONCLUSIONS

Cluster randomized trials play an important role in the development of interventions in (mental) health care but are also subject to threats that may impact their validity. In this contribution, we studied the statistical implications of non‐compliance in an extensive simulation studied and did not only consider ITT and IV but also AT and PP. Our study supplements the existing literature and aids empirical researchers in choosing their approach to handle non‐compliance.

We conclude that avoiding non‐compliance is the best. If non‐compliance cannot be avoided, then at least the covariates that influence non‐compliance must be identified, measured, and included as covariates in the statistical model.

We hope the reader will consider this contribution an interesting companion to earlier work of ours where we studied the statistical implications of other threats to cluster randomized trials: covariate imbalance, contamination, and ignoring a level of nesting.^58^, ^59^, ^60^

## Supporting information

SIM_8351‐Supp‐0001‐Supplementary material ‐ extensions to Figure 2.pdf

SIM_8351‐Supp‐0002‐Supplementary material ‐ extensions to Figure 3.pdf

SIM_8351‐Supp‐0003‐Supplementary material ‐ extensions to Figure 4.pdf

SIM_8351‐Supp‐0004‐Supplementary material ‐ extensions to Figure 5.pdf
