# Supplementary material for: What are the statistical implications of treatment non‐compliance in cluster randomized trials: A simulation study
Source: Stat Med. 2019 Oct 3;38(26):5071–84. doi: 10.1002/sim.8351 (PMC6856967; doi:10.1002/sim.8351)
Supplement: Supplementary file 2 — SIM_8351‐Supp‐0002‐Supplementary material ‐ extensions to Figure 3.pdf [file SIM-38-5071-s002.pdf]

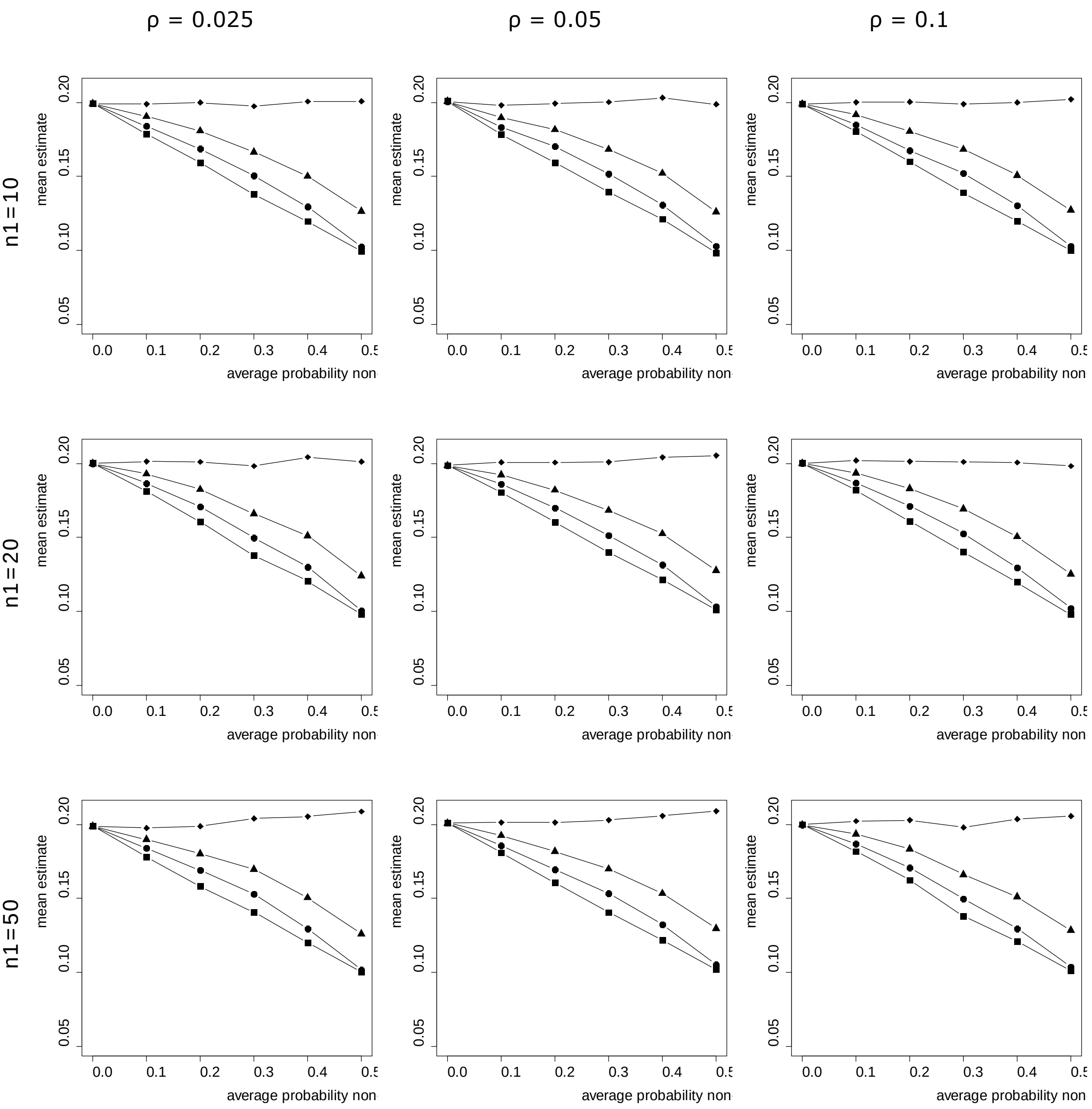

Figure S3a.

Mean estimate as a function of the probability non-compliance for various values of the intraclass correlation coefficient and cluster size. The non-compliance is at the cluster level. The covariate X is not included in the model.

■ = intention to treat; ● = as treated; ▲ = per protocol; ♦ = complier average causal effect

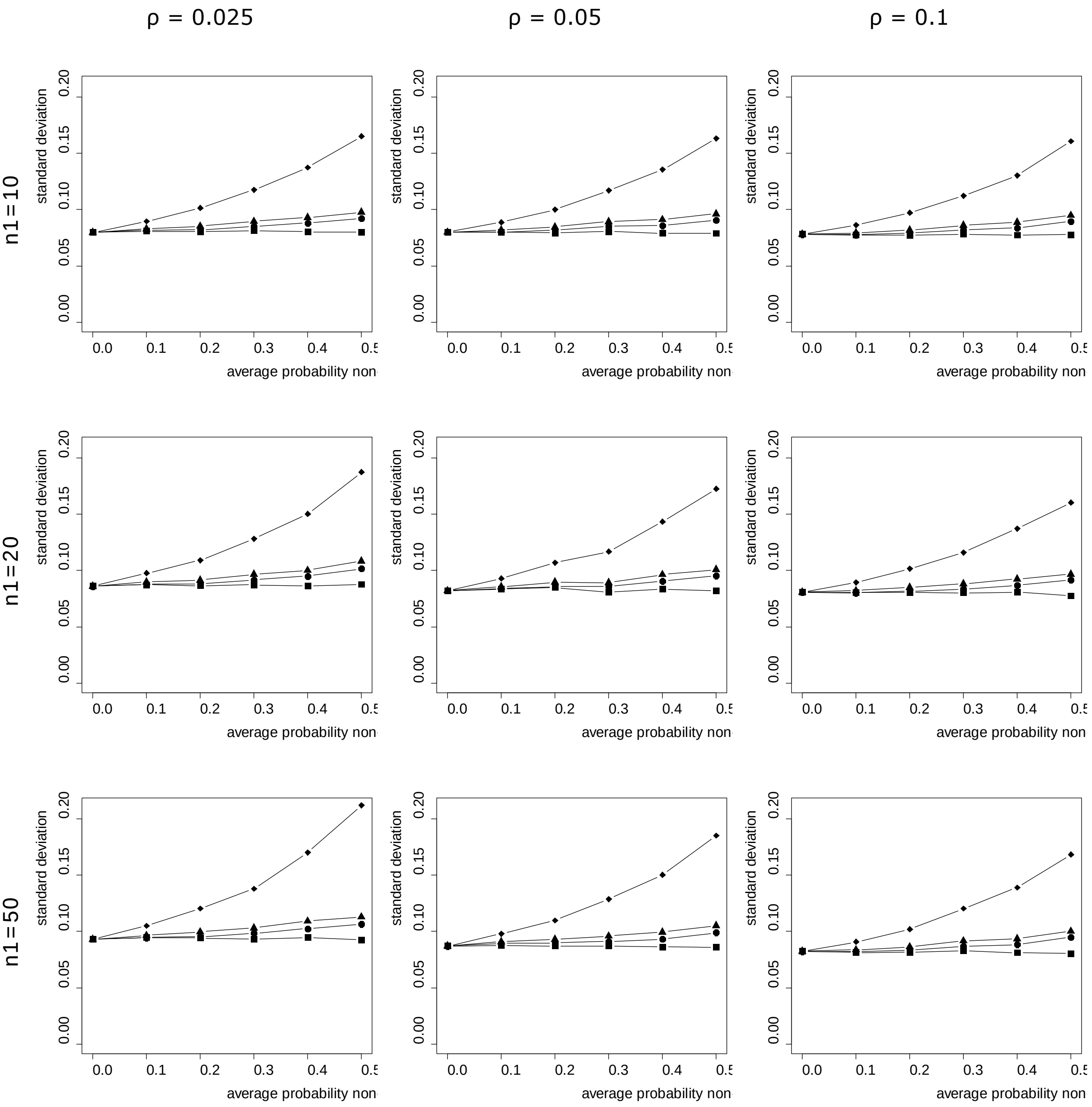

Figure S3b.

Standard deviation as a function of the probability non-compliance for various values of the intraclass correlation coefficient and cluster size. The non-compliance is at the cluster level. The covariate X is not included in the model.

■ = intention to treat; ● = as treated; ▲ = per protocol; ♦ = complier average causal effect

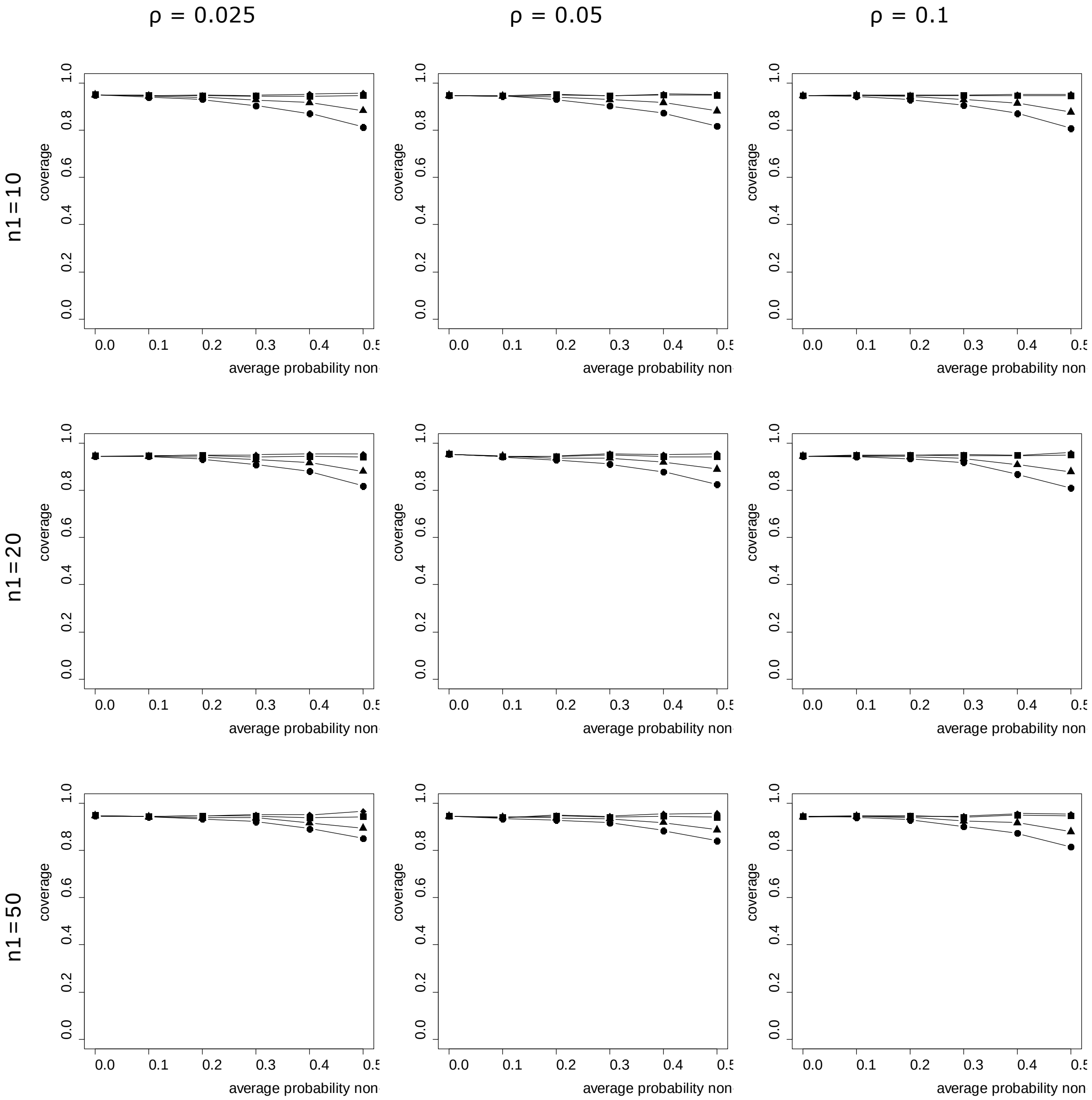

Figure S3c.

Coverage of confidence intervals as a function of the probability non-compliance for various values of the intraclass correlation coefficient and cluster size. The non-compliance is at the cluster level. The covariate X is not included in the model.

■ = intention to treat; ● = as treated; ▲ = per protocol; ♦ = complier average causal effect

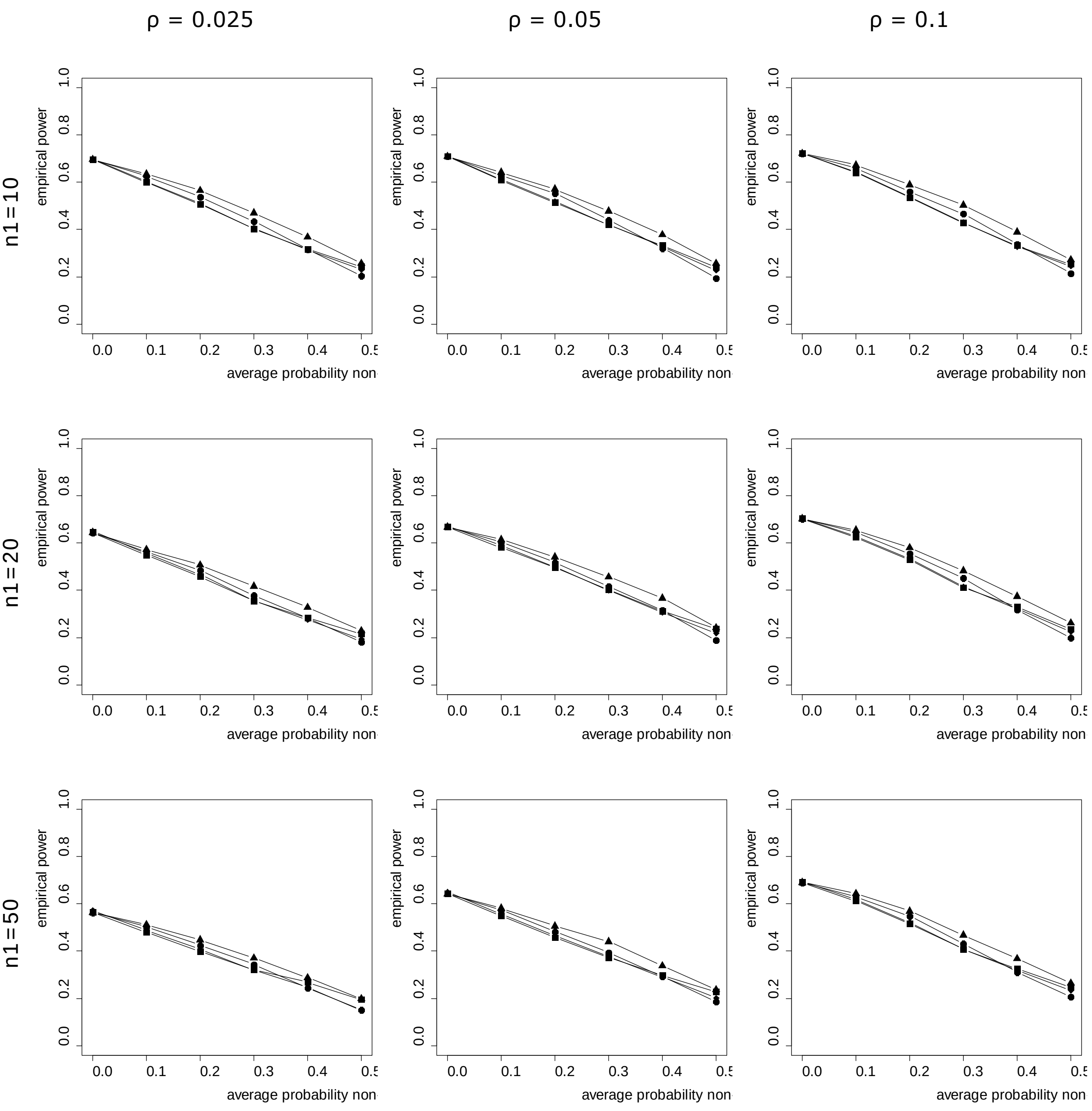

Figure S3d.

Empirical power as a function of the probability non-compliance for various values of the intraclass correlation coefficient and cluster size. The non-compliance is at the cluster level. The covariate X is included not in the model.

■ = intention to treat; ● = as treated; ▲ = per protocol; ♦ = complier average causal effect
